# Neuroplasticity and Neuroprotective Effect of Treadmill Training in the Chronic Mouse Model of Parkinson's Disease

**DOI:** 10.1155/2019/8215017

**Published:** 2019-04-03

**Authors:** Ewelina Palasz, Wiktor Niewiadomski, Anna Gasiorowska, Anna Mietelska-Porowska, Grazyna Niewiadomska

**Affiliations:** ^1^Nencki Institute of Experimental Biology, Polish Academy of Sciences, Warsaw, Poland; ^2^Mossakowski Medical Research Centre, Polish Academy of Sciences, Warsaw, Poland

## Abstract

Physical training confers protection to dopaminergic neurons in rodent models of parkinsonism produced by neurotoxins. The sparing effect of physical training on dopaminergic neurons can be tested with training applied during chronic MPTP treatment, while the neurorestorative effect when training is applied after completing such treatment. In this study, the effect of the onset of training respective to chronic MPTP treatment was specifically addressed. Three groups of mice were injected with 10 doses of MPTP (12.5 mg/kg/injection) over 5 weeks. The first group remained sedentary; the second one underwent early onset training, which started 1 week before commencing MPTP treatment, continued throughout 5 weeks of treatment and 4 weeks thereafter; the third group underwent late-onset training of the same length and intensity as the former group, except that it started immediately after the end of MPTP treatment. Two groups served as controls: a saline-injected group that remained sedentary and saline-injected group, which underwent the same training as the early and late-onset training groups. Both early and late-onset physical training saved almost all nigral and VTA dopaminergic neurons, prevented inflammatory response, and increased the BDNF and GDNF levels to a similar extent. From these results one may conclude that early and late-onset training schedules were equipotent in their neuroprotective effect and that the mechanism of neuroprotection was similar. The sparing effect of early onset training may be satisfactorily explained by assuming that the increased level of BDNF and GDNF prevented the degeneration of dopaminergic neurons. To explain a similar number of dopaminergic neurons detected at the end of the early and late-onset training, one should additionally assume that the former training schedule induced neurogenesis. Results of this study support the view that physical activity may be neuroprotective even at a more advanced stage of PD and justify starting physical activity at any point of the disease.

## 1. Introduction

Since most of the symptoms of Parkinson's disease (PD) are caused by a deficiency of dopamine (DA) in the brain, many drugs are designed to temporarily supplement the level of this neurotransmitter or mimic its action. Currently available treatments are only symptom targeting, their effectiveness decreases with the progression of the disease, and they have many side effects. All these facts highlight the need to seek new treatments supporting the pharmacological therapy of PD. One nonpharmacological approach that supports PD patients is physical exercise. Physical activity alleviates and slows down the development of PD symptoms as demonstrated by improvement in muscle strength, balance, speed and length of stride, daily activities, general wellbeing, and the extended time of independence [1–7]. However, these may be unspecific effects of physical activity, and question arises whether this activity is also a disease-modifying factor. This is suggested by the epidemiological studies, which demonstrated an inverse relationship between physical exercise and the risk of the occurrence and development of PD [8, 9].

The experimental data show that the goal-based exercise and aerobic training can enhance brain plasticity, which plays a key role in improving motor and cognitive functions in people with PD [10]. Current knowledge about the mechanism of the neuroprotective properties of physical training on the functional state of dopaminergic neurons is based on data obtained from animal studies. Many of these studies suggest that exercise can prevent or slow down neurodegenerative processes and rebuild disturbed signaling pathways [11–13]. The neuroprotective effect of physical training is associated with the activation of the neurotrophin signaling pathways [14, 15], synaptogenesis [16], angiogenesis [17] and neurogenesis strengthening [18, 19], reduction of inflammatory processes [20–22], and stabilization of calcium homeostasis [23]. Physical activity prevents the loss of dopaminergic neurons in the substantia nigra pars compacta (SNpc) and also increases the DA level and the sensitivity of its receptor [24, 25]. In the literature, there are a number of reports that physical exercise leads to increased expression of endogenous trophic factors and reduced expression of proinflammatory markers, thereby reducing the vulnerability of dopaminergic neurons to oxidative stress and death. The study performed by Lau et al. [26] has revealed that the neuronal and behavioral recovery produced by exercise in the chronic 1-methyl-4-phenyl-1,2,3,6-tetrahydropyridine (MPTP) mouse model of PD was associated with an improved mitochondrial function and an increase in the brain region-specific levels of brain-derived neurotrophic factor (BDNF) and glial cell line-derived neurotrophic factor (GDNF). In turn, Zhao et al. [27] have shown that vibration training could significantly increase the number of nigrostriatal dopaminergic neurons and the levels of striatal DA and BDNF in the MPTP mice. Using the 6-hydroxydopamine (6-OHDA) model of PD in rats, Tajiri et al. [25] have demonstrated the effects of forced treadmill training on the nigrostriatal dopaminergic projection, neurotrophic factors level, and motor skills of experimental animals.

Recent evidence suggests that physical activity can lead to the reduction of inflammatory processes in the brain of PD patients in the course of neurodegenerative diseases [28]. In a progressive MPTP mouse model of PD, Sconce et al. [20] have noticed recovery of motor skills, increased vesicular monoamine transporter 2 (VMAT2) expression, decreased glycosylated dopamine transporter (DAT) expression, reduced levels of vesicular glutamate transporter 1 (VGLUT1), glutamate transporter-1 (GLT-1), and lower levels of the inflammatory marker, component 3 of the nuclear factor of activated T-cells (NFATc3), and of the astrocytic marker, glial fibrillary acidic protein (GFAP), in MPTP/exercised mice as compared to MPTP mice without exercise. In turn, a four-week swimming training attenuated motor and cognitive impairment, prevented the increase in reactive oxygen species (ROS) and interleukin 1 beta (IL-1*β*) levels, glutathione S-transferase (GST), and glutathione reductase (GR) activity, prevented the inhibition of glutathione peroxidase (GPx) activity, restored the levels of DA and its metabolites in the striatum of mice administered with 6-OHDA [21]. Similarly, Jang et al. [29] have noticed that 8-week treadmill training after chronic MPTP administration in mice restores motor coordination abilities, increases tyrosine hydroxylase (TH) level in the striatum and SNpc, and decreases *α*-synuclein expression in the striatum resulting in downregulation of toll-like receptor 2 (TLR2) signaling molecules such as myeloid differentiation primary response 88 (MyD88), tumor necrosis factor receptor-associated factor 6 (TRAF6), and phosphorylated transforming growth factor-*β*-activated protein kinase 1 (p-TAK1).

In animal models of PD, the neuroprotective effect of physical exercise was examined when training was applied before, during, and after parkinsonism inducing treatment. In addition, these studies have used an acute [25, 30–33] or chronic regime of neurotoxin administration [34]. The studies of Ahmad et al. [34] and Lau et al. [26] demonstrated that physical exercise applied during application of the MPTP neurotoxin prevents, at least partly, the loss of TH-positive nigrostriatal neurons and likely those in the ventral tegmental area (VTA), while the same dose and duration of neurotoxin administration causes moderate loss of these neurons in sedentary mice. Studies on the restorative effect of exercise applied after neurotoxin application show disparate results: from no effect to partial or complete recovery of the number of TH-positive nigrostriatal neurons.

Although these data suggest that physical exercise is effective regardless of whether it starts, there are no data that compare the effectiveness of different physical training onsets. Thus, the aim of this study was to examine precisely the effect of only one factor: the timing of exercise application with respect to the intoxication period. For this purpose, the effects of treadmill training applied before, during, and after induction of the chronic model of PD were compared with the effects of treadmill training of the same intensity and duration applied after completing the MPTP treatment. We applied the chronic PD model in mice [34] in which the induction of parkinsonism takes 5 weeks, during which time 10 injections of MPTP are administered. Such treatment causes neurological deficits resembling PD. Unlike the most commonly used acute and subacute MPTP treatments, after which neurological and behavioral deficits soon spontaneously reverse, the effects of this chronic PD model last at least 6 months. To elucidate the role of exercise alone, training was applied in non-MPTP-treated mice. In order to gain insight into the mechanisms of neuroprotection and neurorestoration, the levels of neurotrophic factors and markers of inflammation were examined. To our knowledge, such comparison of the effect of exercise timing relative to MPTP treatment has not been performed in one and the same study yet.

## 2. Methods

### 2.1. Animals and Treatments

All animal experimental procedures used in this study were approved by the First Warsaw Local Ethics Committee for Animal Experimentation and carried out in accordance with the Polish Law on the Protection of Animals and National Institute of Health's Guide for Care and Use of Laboratory Animals (publication no. 85-23, revised 1985) and the European Union Council Directive (63/2010/EU). Studies were performed on 12-week-old male C57BL/6J mice purchased from the Medical University of Bialystok (Poland) and delivered to the Nencki Institute of Experimental Biology 1 month prior to experiments. The mice were housed at a 12 : 12 h dark light cycle, with constant temperature and humidity (23 ± 1°C, 55 ± 5%), and had free access to both food and water. For the chronic paradigm of MPTP administration, mice received 10 subcutaneous (s.c.) doses of MPTP (12.5 mg/kg in saline; Santa Cruz Biotechnology, Cat. No. sc-206178; Axon Medchem, Cat. No. 1075) in combination with intraperitoneal (i.p.) injections of probenecid (250 mg/kg in dimethyl sulfoxide, DMSO; Sigma-Aldrich, Cat. No. P8761) for 5 weeks [34]. Control mice received saline and DMSO injections only. Mice were subdivided into a control group (C, *n* = 15), control treadmill training group (CTT, *n* = 11), MPTP-injected group (M, *n* = 15), MPTP-injected trained group, which started treadmill training 1 week before the induction of parkinsonism (METT, MPTP + early onset treadmill training, *n* = 13), and MPTP-injected trained group, which started treadmill training immediately after the induction of parkinsonism (MLTT, MPTP + late-onset treadmill training, *n* = 13).  Table 1 presents the experimental groups used in the study. Animals from the groups assigned to treadmill training performed exercises 40 min/day, 5 days/week for 10 weeks. The 40-minute long treadmill protocol consisted of 5 steps: 5 minutes at 10 cm/s, 5 minutes at 15 cm/s, 20 minutes at 20 cm/s, 5 minutes at 25 cm/s, and 5 minutes at 20 cm/s [34].

### 2.2. Immunohistochemistry

The brains of five animals from each group were used for immunohistochemical staining. The animals were deeply anesthetized with Vetbutal and transcardially perfused with cold (4°C) phosphate-buffered saline (PBS) containing 5 IU of heparin per 1 ml of buffer, followed by 4% paraformaldehyde (PVA) in PBS and 5% glycerol with 2% DMSO in PBS. The brains were removed, placed for 1 hour (h) in 4% PVA, and then immersed for cryoprotection in 10% glycerol with 2% DMSO (24 h) and subsequently in 20% glycerol with 2% DMSO (24 h). Forty *μ*m thick frozen sections were washed in PBS (3 × 5 min), incubated with 1% H_2_O_2_ for 30 min at room temperature (RT) to block endogenous peroxidases, and blocked for 1 h at RT in a 5% normal serum solution (NRS). Sections were then incubated with primary antibody diluted in PBS containing 1% bovine serum albumin (BSA), 0.3% Triton X, 5% NRS for 1 h at RT, and overnight at 4°C. In the subsequent step, sections were washed in PBS (3 × 5 min) and incubated for 1 h at RT with secondary antibody diluted to working concentrations in PBS with 5% NRS, 1% BSA, and 0.3% Triton X-100 (see Table 2). Incubation with fluorescent antibodies was carried out in the dark to prevent photobleaching. When 3,3′-diaminobenzidine (DAB, 0.025%) was used as a chromogen, visualization was preceded by a 1-hour incubation at RT with Vectastain ABC kit (Vector Laboratories). Sections were mounted onto slides using UltraCruz® Aqueous Mounting Medium with 4′,6-diamidyno-2-fenyloindol (DAPI, Santa Cruz Biotechnology) or DePeX (SERVA Electrophoresis GmbH) for fluorescent and enzymatic labels, respectively.

### 2.3. Densitometric Analysis of Dopaminergic Neurons

Sections were imaged using a Nikon Eclipse Ni-E microscope. The computerized densitometric image analysis (NIS-Elements BR4.30.00, Nikon Instruments) of TH-immunoreactive (-ir) neurons was performed in SNpc (bregma −3.15 to −3.51) and VTA (bregma −2.79 to −3.07) identified in accordance with the Mouse Brain Stereotaxic Atlas [35]. Regions of interest were outlined using the software's X-Y plotting system that measures the square area (mm^2^) of the marked frame, and TH-ir neurons were counted at 400x magnification. The following criteria were used in the quantitative analysis: neuronal somata were TH-ir, and the cell nucleus and the proximal segment of one or two dendrites was well visible within the counting frame. These criteria enabled the exclusion of noncomplete remnants of neurons. Cell counts per section were then corrected with Abercrombie's formula [36], and the packing density of dopaminergic neurons was calculated as a function of the rostrocaudal level and of location within the VTA and SNpc by using the obtained cell counts and the square area of the marked frames in each analyzed section.

### 2.4. Enzyme-Linked Immunosorbent Assay

Mice were sacrificed by cervical dislocation, the brains were rapidly removed, and the striatum and midbrain containing substantia nigra (SN) were dissected. Tissue samples were weighed and homogenized in 20 volumes of ice-cold homogenization buffer to wet tissue weight. After centrifugation, protein concentrations in the supernatants were determined using Protein Assay Dye Reagent Concentrate (Bio-Rad). Enzyme-linked immunosorbent assay (ELISA) was used to quantify IL-1*β* (ab100705, Abcam), GFAP (NS830, Merck), BDNF (CYT306, Merck), and GDNF (e0043m, EIAab) concentrations in the brain regions of interest. All steps of quantification were performed according to the manufacturer's recommendations. Standards and samples were added to the microtiter plate in triplicate, the optical density of each well was determined at 450 nm (Thermo LabSystems, Multiskan RC Microplate Reader), and protein concentrations were calculated from the standard curve. Results were expressed in pg/ml for BDNF, GDNF, and Il-1*β* or in ng/ml for GFAP.

### 2.5. Statistical Analysis

All data sets are expressed as group mean ± SD. A comparison between experimental groups was carried out with 1-way or 2-way analysis of variance (ANOVA) followed by post hoc comparison using Newman-Keuls test. The differences between groups were considered statistically significant for *p* ≤ 0.05 or below. Statistical analysis was performed using STATISTICA 12 software (StatSoft Polska, http://www.StatSoft.pl).

## 3. Results

### 3.1. The Effect of Chronic MPTP Administration and Physical Training on Dopaminergic Neurons Plasticity

**Figure 1:**
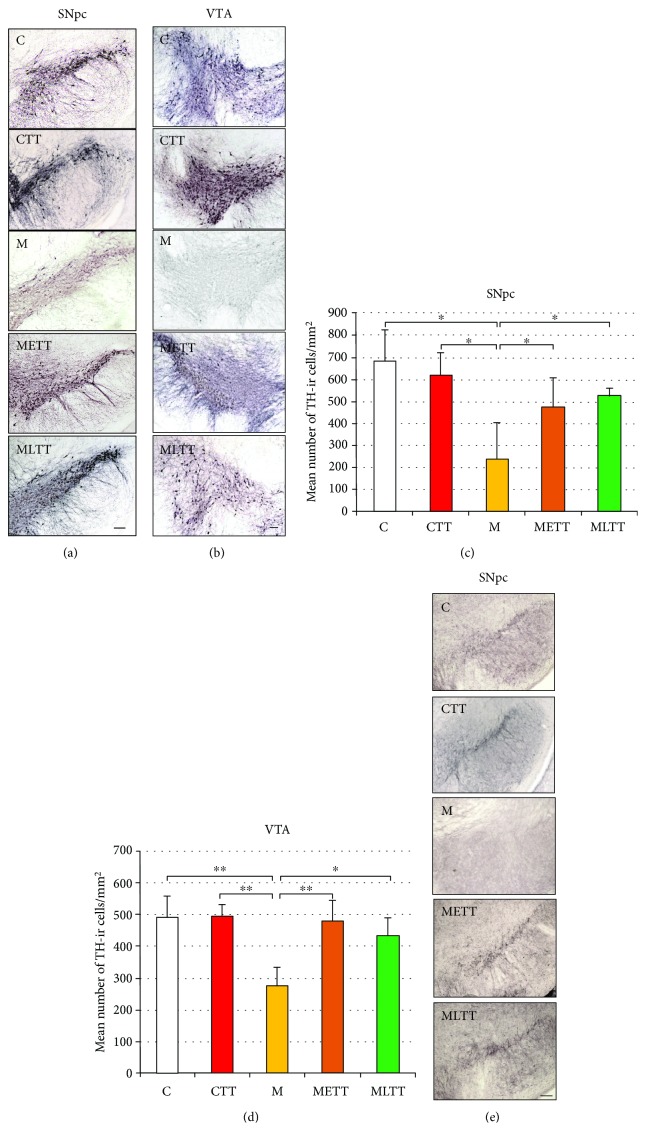
Packing density of TH-immunoreactive neurons in the midbrain regions in the five experimental groups. Representative microphotographs of TH immunohistochemical staining in the substantia nigra pars compacta (SNpc) (a) and ventral tegmental area (VTA) (b), mean packing density of TH-immunoreactive neurons in the SNpc (c) and VTA (d), and microscopic images of VMAT2 immunohistochemical staining in the SNpc (e). C: control; CTT: control + treadmill training; M: treatment with MPTP; METT: MPTP treatment + early onset treadmill training; MLTT: MPTP treatment + late-onset treadmill training group. Scale bar: 100 *μ*m. Statistical comparisons were performed with two-way ANOVA followed by Newman-Keuls *post hoc* test; ^∗∗^*p* < 0.001, ^∗^*p* < 0.05.

TH is the first and rate-limiting enzyme involved in the biosynthesis of catecholamines from tyrosine. From this point, TH is considered as a useful marker of dopaminergic neurons. In order to calculate the number of TH-ir neurons, the brain sections of animal groups, C (*n* = 5), CTT (*n* = 4), M (*n* = 5), METT (*n* = 3), and MLTT (*n* = 3), were used for anti-TH immunohistochemical staining. The focus was placed on the SNpc and VTA, structures where the dopaminergic neurons are located. A substantial reduction in TH staining intensity was observed in both SNpc (Figure 1(a)) and VTA (Figure 1(b)) of MPTP-treated mice (M group) when compared to any other examined group. In addition, a computer-assisted densitometric analysis of packing density of dopaminergic neurons in these structures was performed separately for the left and right hemisphere. In all experimental groups, there were no significant differences in the mean number of TH-ir neurons between the two hemispheres, therefore Figures 1(c) and 1(d) present averaged data of both hemispheres. The densitometric analysis showed a 65% decrease in the number of TH-positive neurons in the SNpc (Figure 1(c); two-way ANOVA, *p* < 0.05) and a 45% decrease in the VTA (Figure 1(d); two-way ANOVA *p* < 0.001) in animals injected with MPTP compared to the control group. Reduction in the number of dopaminergic neurons in the SNpc and VTA of MPTP-treated mice is comparable to the extent of degeneration of dopaminergic neurons observed in the human brain of PD patients. In turn, in both MPTP groups with treadmill training, there was an increase in the number of TH-positive neurons in the SNpc and VTA compared to MPTP sedentary animals. Mice that started training on the treadmill 1 week before the induction of parkinsonism (METT group) showed a 50% increase in TH-positive neurons in the SNpc and 42% in the VTA, while mice that started training on the treadmill shortly after the induction of parkinsonism (MLTT group) showed 55% increase in the number of dopaminergic neurons in the SNpc and 38% in the VTA compared to sedentary MPTP animals (group M).

A 10-week treadmill training either used before (METT group) or after (MLTT group) MPTP administration prevented the loss of TH-ir cells in both SNpc and VTA (METT vs. CTT group; MLTT vs. CTT group, Figures 1(c) and 1(d)). There was no difference in the number of TH-ir cells in both analyzed structures (METT vs. MLTT group, Figures 1(c) and 1(d)) depending on whether the treadmill training started before or after MPTP administration. Neuronal function was also assessed using anti-VMAT2 staining. VMAT2, which in addition to TH is considered to be one of the useful markers of dopaminergic neurons, pumps cytosolic dopamine into synaptic vesicles in an ATP-dependent way. A lower intensity of immunostaining against this protein was found in the SNpc in the M group as compared to the other experimental groups (Figure 1(e)) thus confirming the results obtained for anti-TH staining.

### 3.2. The Effect of Chronic MPTP Administration and Physical Training on the Level of BDNF and GDNF

Neurotrophins, such as BDNF and GDNF, that play an important role in the differentiation, trophic support, and survival of dopaminergic neurons are considered to contribute to the neuroprotective effect of exercise [37–39].

**Figure 2:**
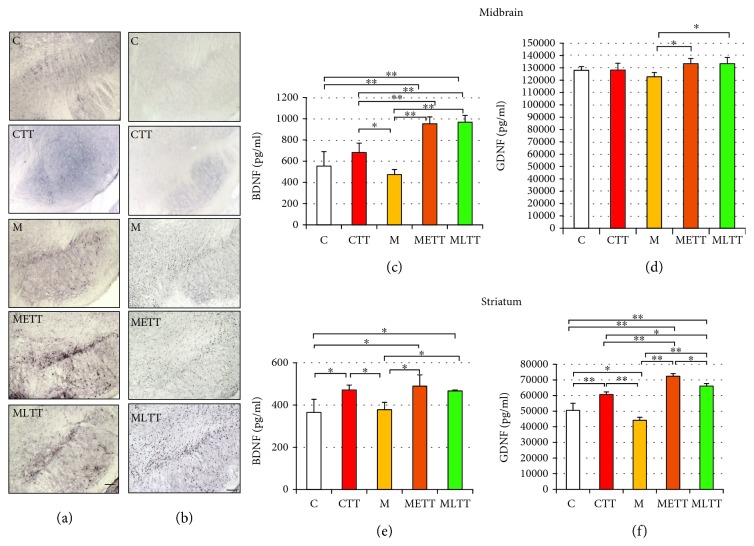
Effect of MPTP and treadmill training on the brain-derived neurotrophic factor (BDNF) and glial cell-derived neurotrophic factor (GDNF) level in the midbrain and striatum. Immunohistochemical staining against BDNF (a) and GDNF (b) in the substantia nigra pars compacta (SNpc), quantitative analysis of BDNF and GDNF in the midbrain (c, d), and striatum (e, f). C: control; CTT: control + treadmill training; M: treatment with MPTP; METT: MPTP + early onset treadmill training; MLTT: MPTP + late-onset treadmill training group. Scale bar: 100 *μ*m. Statistical comparisons were performed with one-way ANOVA followed by Newman-Keuls *post hoc* test; ^∗∗^*p* < 0.001, ^∗^*p* < 0.05.

The impact of physical training and MPTP administration on BDNF and GDNF expression was explored using immunohistochemistry staining against these proteins in the SNpc and an ELISA assay in the midbrain and in the striatum. The immunohistochemical staining in the SNpc showed a significant increase in staining intensity against BDNF (Figure 2(a)) and GDNF (Figure 2(b)) in the METT and MLTT groups as compared to the CTT group as well as to the C group. Additionally, anti-GDNF staining showed a slight increase in intensity in the M group.

ELISA assay revealed significant upregulation of BDNF concentration in the midbrain both in the METT and MLTT groups as compared to the other experimental groups (Figure 2(c), *p* < 0.001) and higher concentration of GDNF in the METT and MLTT groups in comparison to the M group (Figure 2(d), *p* < 0.05). In the striatum, statistical analysis revealed that 10 weeks of treadmill training increased the concentration of BDNF in the CTT group as compared to the C and M groups (Figure 2(e), *p* < 0.05). The physical training accompanied by MPTP administration (METT and MLTT groups) increased also the BDNF concentration in comparison with the C and M groups (Figure 2(e), *p* < 0.05). In respect to GDNF, an increase in its concentration in the CTT group as compared to the C and M groups (Figure 2(f), *p* < 0.001) and opposite direction of GDNF concentration change between groups C and M was observed (Figure 2(f), *p* < 0.05). In addition, GDNF concentrations in both METT and MLTT groups were significantly higher than in other experimental groups (Figure 2(f)). The highest concentration of GDNF was found in the METT group; it was even significantly (*p* < 0.05) higher than the GDNF concentration in the MLTT group (Figure 2(f)).

### 3.3. Reactivity of Astrocyte and Microglia in Response to Chronic MPTP Administration and Physical Training

Glial cells play an important role in maintaining homeostasis of the central nervous system (CNS); however, under certain conditions, long-term stimulation of microglia may activate astrocytes leading to chronic neuroinflammation or even to neurodegeneration. Several studies have highlighted the role of glial cells in the toxic mechanism of MPTP [40–42].

**Figure 3:**
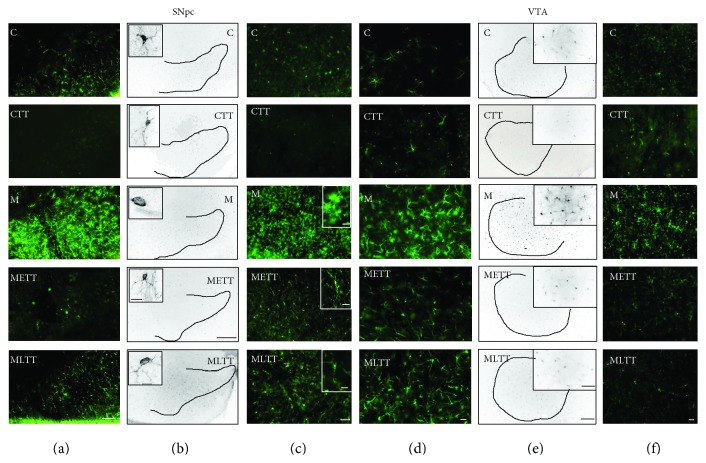
Immunohistochemical staining against the glial fibrillary acid protein (GFAP) (a, d), ionized calcium binding adaptor molecule 1 (Iba1) (b, e), and integrin CD 11b (c, f) in the SNpc (a, b, c) and VTA (D, E, F). C: control; CTT: control + treadmill training; M: treatment with MPTP; METT: MPTP + early onset treadmill training; MLTT: MPTP + late-onset treadmill training group. Scale bar: 200 *μ*m (a); 400 *μ*m and 10 *μ*m (insertions) (b); 50 *μ*m and 10 *μ*m (insertions) (c); 100 *μ*m (d, f). 400 *μ*m and 100 *μ*m (insertions) (e).

To focus on the neuroinflammatory profile induced by MPTP treatment and hypothetical neuroprotective properties of treadmill training on the functioning of dopaminergic neurons, an immunohistochemical analysis of GFAP, Iba1, integrin CD11b in the SNpc and VTA, and immunoenzymatic analysis of GFAP and Il-1*β* in the midbrain and striatum were performed. Microscopic analysis showed a significant increase in the intensity of staining against GFAP (Figures 3(a) and 3(d)), Iba1 (Figures 3(b) and 3(e)), and CD11b (Figures 3(c) and 3(f)) in the SNpc (Figures 3(a)–3(c)) and VTA (Figures 3(d)–3(f)) in the M group compared with the C group. It seems that only in the M group, there was an increase in the number of microglia as a result of their proliferation (Figures 3(b), 3(c), 3(e), and 3(f)). At the same time, the morphological transformation of resting microglia into activated cells which resembled amoeboid phagocytic cells was visible in the MPTP-treated group (Figures 3(b) and 3(e) insertions, M group). The mobilization of microglia in mice with induced parkinsonism was observed both for anti-Iba1 and anti-CD11b staining. The higher intensity of staining against GFAP (Figures 3(a) and 3(d)) in mice exposed to the MPTP neurotoxin indicates that increased activation of astrocytes may contribute to the inflammatory response to dopaminergic neurons injury. It seems that in the METT and MLTT groups, physical training mitigates the proinflammatory response of microglia and astrocytes both in the SNpc and in the VTA (Figures 3(a)–3(f)).

**Figure 4:**
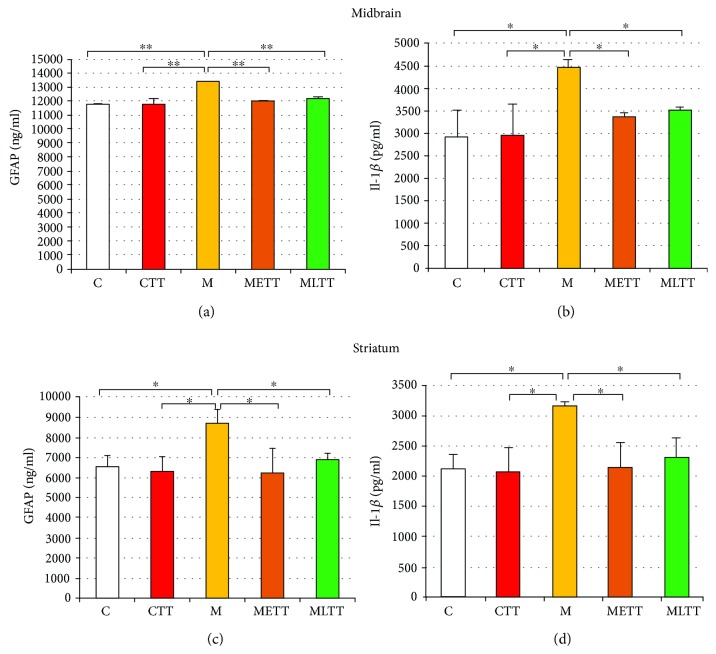
The quantitative analysis of glial fibrillary acid protein (GFAP) (a, c) and interleukin 1 beta (Il-1*β*) (b, d) concentration in the midbrain (a, b) and striatum (c, d). C: control; CTT: control + treadmill training; M: treatment with MPTP; METT: MPTP + early onset treadmill training; MLTT: MPTP + late-onset treadmill training group. Statistical comparisons were performed with one-way ANOVA, followed by Newman-Keuls *post hoc* test; ^∗∗^*p* < 0.001, ^∗^*p* < 0.05.

Quantitative ELISA assessment confirmed data from the microscopic analysis and revealed a significant increase in the level of GFAP and a proinflammatory cytokine Il-1*β* both in the midbrain (Figure 4(a), *p* < 0.001 for GFAP and Figure 4(b), *p* < 0.05 for Il-1*β*) and in the striatum (Figures 4(c) and 4(d), *p* < 0.05 for GFAP and Il-1*β*) in the M group as compared to the rest of the experimental groups. The level of GFAP and Il-1*β* in groups subjected to intoxication and training did not differ significantly from the level of both proteins in the sedentary and training control groups (Figures 4(a) and 4(d)).

## 4. Discussion

The present work compared the effect of long-term physical activity initiated before and after parkinsonism induction on the number of midbrain dopaminergic neurons, the level of neurotrophic factors, and inflammatory process in dopaminergic structures in a chronic MPTP mouse model of PD.

### 4.1. The Neuroprotective Effects of Treadmill Training on Dopaminergic Neurons

It has long been recognized that physical activity tends to have a positive effect in humans in the context of motor skills and proper functioning of the CNS, including the influence on cognitive functions. There is also an increasing body of evidence that physical exercises help recovery and lessen the risk of CNS damage and development of neurodegenerative diseases in animal models. Although there are reports in the literature on the neuroprotective effects of various forms of physical exercise of different duration and intensity on the state of dopaminergic neurons, there is still no definite answer to the question of which form of physical activity gives the best results. In the conducted study, modified forced treadmill training described by Ahmad et al. [34], and then also verified by Pothakos et al. [43] and Lau et al. [26], was implemented. Such training schedule, in contrast to voluntary training or the enriched environment, provides more comparable experimental conditions. The present study tested the effectiveness of two treadmill training schedules: the first one (i) started one week before the beginning of neurotoxin treatment, lasted throughout the 5 weeks of neurotoxin administration, and was continued 4 weeks after neurotoxin treatment (preceding training), while the second one (ii) started immediately after 5 weeks of neurotoxin treatment and lasted 10 weeks (follow-up training). It was found that 10 weeks of exercise, applied both before and after induction of parkinsonism, effectively protects dopaminergic neurons in the SNpc and VTA against the toxic action of MPTP.

It has been shown in animal models of PD that physical exercise is neuroprotective when applied before, during, and after parkinsonism inducing treatment. This means that physical exercise may have preventive, protective, or restorative effects, respectively. The preventive effect of exercise was shown by Gerecke et al. [31] who found that intensive exercise on the running wheel performed by mice for 3 months before acute administration of MPTP completely prevented the loss of dopaminergic neurons in the SNpc and that less intensive exercise or/and performed for a shorter time produced only partial neuroprotection or no protection at all. However, complete neuroprotection was not accompanied by a complete recovery of DA level in the striatum. Fisher et al. [30] found that 30 days of treadmill training in C57 BL/6J mice applied 4 days after acute MPTP administration resulted in a significant downregulation of striatal DAT in the MPTP-treated exercised mice compared to MPTP-treated nonexercised mice but in no significant difference in the TH protein levels. No observation on the number of DA nigrostriatal neurons has been reported. Kintz et al. [32] found that 37 days of exercise starting 5 days after acute MPTP administration did not increase DA striatal level reduced by this neurotoxin. Zhao et al. [27] have shown that vibration training in mice, applied after 1 week of MPTP treatment and lasting 4 weeks, brought the number of nigrostriatal dopaminergic neurons almost to the level noted in control mice and significantly above that in MPTP-treated nontrained mice. A similar result was obtained for striatal DA level. Jang et al. [33] found that 6 weeks of treadmill training applied four weeks after one week lasting MPTP treatment (25 mg/kg daily) resulted in a number of TH-positive neurons almost equal to that in control mice and in the recovery of the TH and DAT levels. Also, in a rat model, Tajiri et al. [25] found significant preservation of TH-positive fibers in the striatum and TH-positive neurons in the SNpc caused by 4 weeks of treadmill training applied 24 hours after a 6-OHDA lesion of right striatum of female rats.

In order to examine the bona fide neuroprotective role of exercise, training should be applied concomitantly with induction of parkinsonism. Such possibility is offered by the chronic PD model in mice, in which induction of parkinsonism takes 5 weeks, during which time 10 injections of MPTP are administered. Such treatment causes neurological deficits showing many features resembling PD [44]. Ahmad et al. [34], using this chronic model of PD, have demonstrated that the treadmill training starting one week before, continued over 5 weeks of MPTP treatment and 4 or 12 weeks thereafter, protected dopaminergic neurons in the VTA. Shorter training produced a significant although a small increase in the number of VTA TH-positive cells comparing to sedentary MPTP mice, whereas longer training returned their number to that observed in the nonparkinsonian control mice. Pothakos et al. [43] using a similar chronic model and starting treadmill training one week before MPTP treatment, maintaining it during 5 weeks of intoxication and 8-12 weeks thereafter, found neither reduction in depletion of striatal DA nor sparing of TH-positive neurons in the SNpc in MPTP-treated exercising mice vs. MPTP-treated sedentary ones. Of possible importance, the difference between these two studies was that the MPTP dose in the latter study was twice that used in the former one, i.e., 12.5 vs. 25 mg/kg/injection. The significance of the dose seems to be confirmed by Lau et al. [26], who used the same chronic model and 18-week treadmill training starting 1 week before the commencement of MPTP treatment. In contrast to Pothakos et al. [43], they observed a significant though incomplete recovery of the number of TH-positive neurons in the SNpc and, similarly, a significant though incomplete restoration of the striatal DA level when compared to that in control mice. The difference in MPTP dosing seems to be reflected by the loss of TH-positive neurons, with a dose of 15 mg MPTP/kg/injection used in Lau et al.'s study [26]; 55% of these neurons were lost vs. 72% in the study of Pothakos et al. In Al-Jarrah et al.'s study [45], four weeks of aerobic training following 5 weeks of chronic MPTP treatment with 25 mg/kg/injection and 1 week of pretraining resulted in a minimal rise of nigrostriatal TH and DA in mice as compared to sedentary MPTP-treated ones. Koo et al. [46] have investigated the effects of 8 weeks of progressive treadmill exercise applied 2 weeks after the completion of chronic MPTP treatment. Despite using the 25 mg/kg/injection dose of MPTP, i.e., the same as Pothakos et al. [43] and Al-Jarrah et al. [45], these investigators found a significantly reduced dopaminergic neuron loss in the SNpc and similar restitution of the TH and DAT level in MPTP-treated exercising mice. Of note is the 50% loss of TH-positive neurons in sedentary MPTP-treated mice, closer to that observed by Lau et al. [26] who used 15 mg/kg/injection than to that noted by Pothakos et al. [43] who applied 25 mg/kg/injection.

However, there are also studies in which there was no effect of physical training on TH-positive cell number, despite a marked improvement in motor performance [20, 43, 47]. The inability to observe the neuroprotective effect of physical activity on dopaminergic neurons may be due to insufficient time needed to restore the proper level of dopaminergic neuronal markers or to the experimental design, e.g., the animals used (species, strain, and age), the applied therapeutic intervention method (treadmill vs. wheel running, brief vs. continual, low vs. high intensity, and light vs. dark cycle), the nature of neurotoxin administration, and the type and dose of the neurotoxin used [48].

Although there are a number of reports in the literature about the protective effect of exercise on the reduction of parkinsonian symptoms in animals, there are no data comparing the effectiveness of physical exercise applied before and after PD induction. The present study evaluated the effect of physical exercise depending on the time of its application: before or after induction of parkinsonism. The comparison of both training schedules in one experiment, in which elements of the procedure were comparable, convincingly demonstrated that both early onset and late-onset physical training exert a beneficial effect on the survival and/or restoration of midbrain dopaminergic neurons, as well as on the expression of trophic factors and the degree of inflammation in the brain in response to the toxic action of MPTP. It is very intriguing that the training applied after intoxication was as advantageous as the training preceding the induction of parkinsonism. Emphasizing this point may bring about substantial progress in the clinical treatment of PD patients with physical exercises as a complementary therapy.

### 4.2. Training on the Treadmill Raises the Level of Neurotrophins

Since the reduction of the BDNF [49] and GDNF [50] levels in PD brains has been observed, a new concept has emerged that an increase in the concentration of neurotrophic factors, which are considered to be capable of supporting neuronal survival, may be neuroprotective in PD [51, 52]. In recent years, a number of clinical trials have been carried out by directly injecting neurotrophins into the brain and implanting genetically modified neurotrophin-producing cells or gene vectors to protect dopaminergic neurons [53]. It seems, however, that less invasive methods, such as long-term physical exercise, may also be neuroprotective and neurorestorative and lead to increased levels of endogenous neurotrophic factors.

In the present study, the effect of 10 weeks of treadmill training, starting both before and after MPTP administration, resulted in an increase of BDNF concentration in the midbrain and in the striatum compared to MPTP-treated sedentary mice. Furthermore, an increase in BDNF in the midbrain in both MPTP groups with treadmill training was also observed, compared to sedentary control and control with treadmill training, and in the striatum compared to sedentary control. It seems that the effect of training was potentiated by MPTP treatment. This phenomenon is absent in the striatum, wherein training, irrespective of whether accompanied by MPTP treatment or not, increases significantly and to a similar degree, the BDNF levels in CTT, METT, and MLTT groups.

Changes in the GDNF levels in the striatum strongly resemble those of the BDNF levels in the midbrain. There is also evident potentiation of the effect of training by MPTP treatment. However, late-onset training caused a somewhat smaller, though statistically significant, rise of the GDNF level than the early onset one. The GDNF levels in the midbrain are slightly elevated by early onset and late-onset training and significantly higher only in comparison with sedentary MPTP-treated mice. These observations correspond with those presented by Lau et al. [26]. In their study, the effects of chronic treatment with MPTP were compared in sedentary vs. early trained mice, i.e., 18 weeks of training starting 1 week before the 5 weeks of MPTP (15 mg/kg/injection) treatment. As in our study, this kind of training significantly rises BDNF in the SNpc and GDNF in the striatum as compared to control and mice chronically treated with MPTP. Training also raises the striatal BDNF levels, but the increase does not attain statistical significance, and also increases the nigral GDNF level slightly but significantly above that in sedentary chronically MPTP-treated mice. Qualitatively, the results of Lau et al. and ours agree. Moreover, in our study, in contrast to Lau et al.'s study, there is a training control group; thus, the effects of exercise alone can be elucidated.

An increased midbrain level of BDNF, induced by training, may have a protective role on dopaminergic neurons as normalization of the striatal TH and DA level was observed in parkinsonian MPTP mice [26]. In turn, Tajiri et al. [25], using rats with 6-OHDA lesion, also demonstrated an effect of treadmill training on the upregulation of the BDNF and GDNF level in the striatum of the exercise group. Available literature provides several additional arguments supporting the thesis of the key role played by BDNF and GDNF in dopaminergic neuroprotection. Boger et al. [54] have observed an accelerated and age-related decrease in TH immunostaining in the SN in GDNF+/- mice as compared to control animals. In turn, Gerecke et al. [55] have demonstrated that BDNF+/− mice allowed 90 days of unrestricted exercise were not protected from MPTP-induced dopaminergic neuron loss in the SNpc, while control mice allowed 90-day exercise demonstrated complete protection against MPTP-induced neurotoxicity. Furthermore, blocking the action of BDNF, using tropomyosin receptor kinase B (TrkB) antagonist, suppressed exercise-induced protection against lipopolysaccharide- (LPS-) induced damage to dopaminergic neurons [56]. A similar conclusion was made by Real et al. [57], who found that the neuroprotective effect of physical training in the 6-OHDA rat model was not observed when a blocker of BDNF receptors was used. These results suggest that physical activity may be neuroprotective and may reduce the sensitivity of dopaminergic neurons to toxins by activating signaling cascades triggered by the increased availability of BDNF and GDNF.

### 4.3. Physical Training Attenuates the Inflammatory Process Induced by Chronic MPTP Administration

Although the etiology of Parkinson's disease is not fully understood, chronic inflammation plays an important role in the development of PD. On the other hand, it is not yet clear whether inflammation is a primary or secondary phenomenon that is a consequence of neuronal death [58]. Characteristic features of neuroinflammation include activated microglia and reactive astrocytes known to produce cytokines, chemokines, prostaglandins (PG), protein complement cascades, ROS, and reactive nitrogen species (RNS) [59]. The immunofluorescence staining against GFAP, in the presented study, showed an increased number of astrocytes in SNpc and VTA in MPTP sedentary mice as compared to all other groups. GFAP concentration was also verified using the ELISA method. The ELISA results were consistent with the immunohistochemical GFAP staining, i.e., the highest GFAP concentration was observed in the MPTP group. These observations concerned both midbrain and striatum. On the other hand, treadmill training caused reduced GFAP concentration in both MPTP-trained groups (METT and MLTT), comparable to the concentration of GFAP observed in the control groups (C and CTT). Similar results were described by Sconce et al. [20], who showed an increased GFAP level in the SN in animals treated with an increasing dose of MPTP for 4 weeks. The difference in results obtained by Sconce et al. and those presented in this study was that the group treated with MPTP and exercised had an elevated GFAP level compared to the control group; however, it was still much lower than in the MPTP sedentary animals. Furthermore, staining against CD11b and Iba1, markers of microglia, also showed higher intensity in SNpc and VTA in MPTP mice without treadmill training compared with the results obtained in controls and both MPTP groups with treadmill training. Additionally, higher magnification of anti-Iba1 staining in the SNpc indicated a change of microglial cell phenotype from resting to amoeba-like proinflammatory shape.

It seems that during the development of neurodegenerative diseases, a proinflammatory way of activation plays a pivotal role. The mutual activation of microglia and astrocytes depends mainly on the inflammatory cytokines secreted by them or the interaction of their receptors [60]. In addition, it was shown that cytokines secreted from activated microglia can lead to activation of astrocytes, which are cytotoxic to neurons, and these reactive astrocytes potentiate ongoing inflammation [60, 61]. Il-1*β* and/or TNF-*α*, key proinflammatory cytokines, are considered to be implicated in the early signaling pathways leading to astrogliosis [62]. Although Il-1*β* is secreted mainly by activated microglia [60], reactive astrocytes also show Il-1*β* expression [63]. In the present study, it was found that concentration of Il-1*β* was elevated in the MPTP sedentary group, both in the midbrain and in the striatum, while Il-1*β* concentration in MPTP mice with early and late training was similar to that observed in the control groups. These results suggest that MPTP administration leads to activation of the proinflammatory phenotype of glial cells and that physical training alleviates this activation and may inhibit the inflammatory process within the brain. The hypothesis that the inflammatory process is involved in the degeneration of dopaminergic neurons and is associated with the administration of MPTP and that treadmill exercise inhibits the activation of microglial cells in treated mice was previously demonstrated by another research groups [64].

Both subtypes of glial cells, astrocytes and microglia, may be activated in two different ways, resulting in the formation of proinflammatory (classical M1 activation) or anti-inflammatory (alternative M2 activation) response. Alternatively stimulated microglia show increased expression of cytokines recognized as anti-inflammatory, such as IL-10, TGF-*β*, IGF-1, NGF, and BDNF [65]. Astrocytes, like microglia, also secrete anti-inflammatory agents into the environment, including neurotrophic factors (e.g., GDNF, BDNF, and MANF (mesencephalic astrocyte-derived neurotrophic factor)), which stimulate the survival and revive damaged dopaminergic neurons [66]. In addition, in *in vitro* conditions, it was shown that endogenous IL-1*β* may induce gene expression, synthesis, and secretion of GDNF [67]. It is possible that physical training applied in MPTP mice with parkinsonism launches an alternative neuroprotective activation of microglia rather than reduces proinflammatory glial activation. Such neuroprotective activation could result from increased synthesis of trophic factors induced by prolonged physical exertion. Then, there is no proinflammatory proliferation and activation of glial cells because dopaminergic neurons, protected by neurotrophins, do not degenerate and do not send signals that mobilize the inflammatory response.

### 4.4. Equipotent Neuroprotective Effect of Early Onset and Late-Onset Training

To our knowledge, this is the first study in which the impact of the timing of physical training on the neuroprotective effect observed in a murine model of PD has been precisely addressed. This was achieved by using the same murine strain, dose, and mode of neurotoxin administration (i.e., chronic treatment with 10 injections of 12.5 mg/kg/injection of MPTP) and the same kind, duration, and intensity of physical effort but different onset of training: the training started either 1 week before and continued over the 5-week period of MPTP treatment and beyond (for 4 weeks) or the training started immediately after the conclusion of the neurotoxin treatment and also lasted 10 weeks.

It was found that both the early onset and late-onset training (1) almost completely preserved the number of dopaminergic neurons in SNpc and VTA, (2) increased to a similar degree the BDNF level in the midbrain and the GDNF level in the striatum, and (3) entirely prevented inflammatory response evoked by chronic MPTP treatment. A possible explanation of the neuroprotective effect of late-onset training should account for the greater number of dopaminergic neurons in late-onset training MPTP-treated mice than in sedentary MPTP-treated mice. Their number should have been similarly reduced in both groups at the end of 5 weeks of chronic MPTP treatment as these groups were treated in the same way during this period. It may be assumed that there was a further decline in the number of dopaminergic neurons in sedentary MPTP-treated mice, during the 4 weeks, from the termination of MPTP treatment to brain isolation. During the same 4 weeks, physical training induced preservation or reduced decline of dopaminergic neurons in late-onset training MPTP-treated mice. This decline of dopaminergic neurons in the former group and their preservation in the latter group would account for the difference in the number of these neurons between groups.

Preservation of dopaminergic neurons might be due to increased midbrain level of BDNF, what in turn prevents the degeneration of these neurons and restrains inflammatory response, as reflected by the diminished level of inflammatory markers in late-onset training MPTP-treated group. This hypothesis implies that degeneration of neurons progresses also after the termination of MPTP treatment. Subsistence of the inflammatory process 4 weeks after the cessation of neurotoxin treatment was confirmed in our study by a significantly higher level of inflammatory markers in sedentary MPTP-treated mice. This is in agreement with others' observations that such degeneration continues 3 weeks after MPTP/probenecid treatment and is accompanied by a significant inflammatory response [68].

Training-induced preservation of dopaminergic neurons may not account for the fact that in early onset training MPTP-treated group, the number of dopaminergic neurons was similar to that in the late-onset training MPTP group. If training exerted its neuroprotective effect already during MPTP treatment, then the number of dopaminergic neurons after cessation of this treatment should be higher in the early onset training MPTP group than that in the late-onset training MPTP group. The sparing effect would be present during 4 weeks following the termination of MPTP treatment, as both groups trained at that time, but preservation alone could not result in a similar number of dopaminergic neurons. Adding increase in number of dopaminergic neurons would explain their similar number in both groups. It is known that spontaneous regeneration of dopaminergic neurons occurs in acute MPTP mice models [69, 70]. The chronic treatment with MPTP, such as applied in this study, is claimed to prevent this regeneration [68]; however, it is possible that also in such model of parkinsonism training leads to recovery by inducing neurogenesis [25, 33] or by restoring neurotransmitter phenotype [71]. In order to make recovery a more plausible explanation of our observations, one can postulate that the time window for this process starts with some delay after the end of MPTP treatment, thus allowing for an increase in a number of dopaminergic neurons during the last 6 weeks of training of late-onset MPTP-treated mice. If neurogenesis and/or restoration of neurotransmitter phenotype takes place, the importance of continued physical activity in PD patients should be strongly underpinned.

## 5. Conclusions

This study supports the view that physical effort is neuroprotective also after the neurotoxic assault. Furthermore, it suggests a similar mechanism of neuroprotection of early and late-onset physical training. Although the actual sequence of events remains to be elucidated, it seems that neurotrophic factors prevent neurodegeneration and in this way prevent the appearance of the inflammatory response. These results underscore the conviction that physical activity may be neuroprotective also at a more advanced stage of PD and justify the rationale of starting physical activity at any point of the disease, as long as it is feasible.

## Figures and Tables

**Table 1:** Experimental groups used in the study.

Group	Description of the treatment	Number of mice (*n*)	Treadmill training	The total duration of the experiment (weeks)
C (Control)	Saline (s.c.) and DMSO (i.p.) injections	15	**NO**	10
CTT (Control + treadmill training)	Saline (s.c.) and DMSO (i.p.) injections, 10 weeks of treadmill training	11	**YES**	10
M (MPTP)	MPTP (s.c.) and probenecid (i.p.) injections	15	**NO**	10
METT (MPTP + early onset treadmill training)	MPTP (s.c.) and probenecid (i.p.) injections 10 weeks of treadmill training started 1 week before the intoxication	13	**YES**	10
MLTT (MPTP + late-onset treadmill training)	MPTP (s.c.) and probenecid (i.p.) injections 10 weeks of treadmill training started immediately after the intoxication	13	**YES**	16

**Table 2:** List of antibodies, their suppliers, and dilutions used in this study.

Primary antibody	Supplier	Primary antibody dilution	Secondary antibody	Supplier	Secondary antibody dilution
Antityrosine hydroxylase (TH, AB152)	Merck	1 : 1000	Biotinylated Goat Anti-Rabbit IgG Antibody (BA-1000)	Vector Laboratories	1 : 200
Antivesicular monoamine transporter 2 (VMAT 2, sc-15314)	Santa Cruz Biotechnology	1 : 200	Biotinylated Goat Anti-Rabbit IgG Antibody (BA-1000)	Vector Laboratories	1 : 200
Antiglial cell-derived neurotrophic factor (GDNF, sc-328)	Santa Cruz Biotechnology	1 : 500	Biotinylated Goat Anti-Rabbit IgG Antibody (BA-1000)	Vector Laboratories	1 : 200
Anti-brain-derived neurotrophic factor (BDNF, sc-20981)	Santa Cruz Biotechnology	1 : 500	Biotinylated Goat Anti-Rabbit IgG Antibody (BA-1000)	Vector Laboratories	1 : 200
Anti-CD11b (MCA711G)	Bio-Rad	1 : 200	Goat anti-Rat IgG (H+L) Cross-Adsorbed Antibody, Alexa Fluor 488 (A-11006)	ThermoFisher	1 : 1000
Antiglial fibrillary acidic protein (GFAP, Z0334)	Dako	1 : 1000	F(ab')2-Goat anti-Rabbit IgG (H + L) Cross-Adsorbed Antibody, Alexa Fluor 488 (A-11070)	ThermoFisher	1 : 1000
Anti-ionized calcium binding adaptor molecule 1 (Iba1, ab5076)	Abcam	1 : 500	Rabbit Anti-Goat IgG Antibody, HRP conjugate (AP106P)	Merck	1 : 500

## Data Availability

The data used to support the findings of this study are available from the corresponding author upon request.

## References

[1] Miyai I., Fujimoto Y., Ueda Y. (2000). Treadmill training with body weight support: its effect on Parkinson’s disease. *Archives of Physical Medicine and Rehabilitation*.

[2] Berendse H. W., Groenewegen H. J. (1990). Organization of the thalamostriatal projections in the rat, with special emphasis on the ventral striatum. *Journal of Comparative Neurology*.

[3] Bergen J. L., Toole T., Elliott RG 3rd, Wallace B., Robinson K., Maitland C. G. (2002). Aerobic exercise intervention improves aerobic capacity and movement initiation in Parkinson’s disease patients. *NeuroRehabilitation*.

[4] Hirsch M. A., Toole T., Maitland C. G., Rider R. A. (2003). The effects of balance training and high-intensity resistance training on persons with idiopathic Parkinson’s disease. *Archives of Physical Medicine and Rehabilitation*.

[5] Reuter I., Mehnert S., Leone P., Kaps M., Oechsner M., Engelhardt M. (2011). Effects of a flexibility and relaxation programme, walking, and Nordic walking on Parkinson’s disease. *Journal of Aging Research*.

[6] Herman T., Giladi N., Gruendlinger L., Hausdorff J. M. (2007). Six weeks of intensive treadmill training improves gait and quality of life in patients with Parkinson’s disease: a pilot study. *Archives of Physical Medicine and Rehabilitation*.

[7] Nadeau A., Pourcher E., Corbeil P. (2014). Effects of 24 wk of treadmill training on gait performance in Parkinson’s disease. *Medicine and Science in Sports and Exercise*.

[8] Chen H., Zhang S. M., Schwarzschild M. A., Hernan M. A., Ascherio A. (2005). Physical activity and the risk of Parkinson disease. *Neurology*.

[9] Yang F., Trolle Lagerros Y., Bellocco R. (2015). Physical activity and risk of Parkinson’s disease in the Swedish National March Cohort. *Brain*.

[10] Petzinger G. M., Fisher B. E., McEwen S., Beeler J. A., Walsh J. P., Jakowec M. W. (2013). Exercise-enhanced neuroplasticity targeting motor and cognitive circuitry in Parkinson’s disease. *Lancet Neurology*.

[11] Ang E.-T., Tai Y.-K., Lo S.-Q., Seet R., Soong T.-W. (2010). Neurodegenerative diseases: exercising towards neurogenesis and neuroregeneration. *Frontiers in Aging Neuroscience*.

[12] Almeida M. F., Chaves R. S., Silva C. M., Chaves J. C. S., Melo K. P., Ferrari M. F. R. (2016). BDNF trafficking and signaling impairment during early neurodegeneration is prevented by moderate physical activity. *IBRO Reports*.

[13] Almeida M. F., Silva C. M., Chaves R. S. (2017). Effects of mild running on substantia nigra during early neurodegeneration. *Journal of Sports Sciences*.

[14] Sleiman S. F., Henry J., al-Haddad R. (2016). Exercise promotes the expression of brain derived neurotrophic factor (BDNF) through the action of the ketone body *β*-hydroxybutyrate. *eLife*.

[15] Phillips C., Baktir M. A., Srivatsan M., Salehi A. (2014). Neuroprotective effects of physical activity on the brain: a closer look at trophic factor signaling. *Frontiers in Cellular Neuroscience*.

[16] Ambrogini P., Lattanzi D., Ciuffoli S., Betti M., Fanelli M., Cuppini R. (2013). Physical exercise and environment exploration affect synaptogenesis in adult-generated neurons in the rat dentate gyrus: possible role of BDNF. *Brain Research*.

[17] Lopez-Lopez C., LeRoith D., Torres-Aleman I. (2004). Insulin-like growth factor I is required for vessel remodeling in the adult brain. *Proceedings of the National Academy of Sciences of the United States of America*.

[18] van Praag H., Shubert T., Zhao C., Gage F. H. (2005). Exercise enhances learning and hippocampal neurogenesis in aged mice. *Journal of Neuroscience*.

[19] van der Borght K., Kóbor-Nyakas D. É., Klauke K. (2009). Physical exercise leads to rapid adaptations in hippocampal vasculature: temporal dynamics and relationship to cell proliferation and neurogenesis. *Hippocampus*.

[20] Sconce M. D., Churchill M. J., Greene R. E., Meshul C. K. (2015). Intervention with exercise restores motor deficits but not nigrostriatal loss in a progressive MPTP mouse model of Parkinson’s disease. *Neuroscience*.

[21] Goes A. T. R., Souza L. C., Filho C. B. (2014). Neuroprotective effects of swimming training in a mouse model of Parkinson’s disease induced by 6-hydroxydopamine. *Neuroscience*.

[22] Real C. C., Garcia P. C., Britto L. R. G. (2017). Treadmill exercise prevents increase of neuroinflammation markers involved in the dopaminergic damage of the 6-OHDA Parkinson’s disease model. *Journal of Molecular Neuroscience*.

[23] Chen W., Qiao D., Liu X., Shi K. (2017). Treadmill exercise improves motor dysfunction and hyperactivity of the corticostriatal glutamatergic pathway in rats with 6-OHDA-induced Parkinson’s disease. *Neural Plasticity*.

[24] Petzinger G. M., Holschneider D. P., Fisher B. E. (2015). The effects of exercise on dopamine neurotransmission in Parkinson’s disease: targeting neuroplasticity to modulate basal ganglia circuitry. *Brain Plasticity*.

[25] Tajiri N., Yasuhara T., Shingo T. (2010). Exercise exerts neuroprotective effects on Parkinson’s disease model of rats. *Brain Research*.

[26] Lau Y.-S., Patki G., Das-Panja K., Le W.-D., Ahmad S. O. (2011). Neuroprotective effects and mechanisms of exercise in a chronic mouse model of Parkinson’s disease with moderate neurodegeneration. *European Journal of Neuroscience*.

[27] Zhao L., He L. X., Huang S. N. (2014). Protection of dopamine neurons by vibration training and up-regulation of brain-derived neurotrophic factor in a MPTP mouse model of Parkinson’s disease. *Physiological Research*.

[28] Gleeson M., Bishop N. C., Stensel D. J., Lindley M. R., Mastana S. S., Nimmo M. A. (2011). The anti-inflammatory effects of exercise: mechanisms and implications for the prevention and treatment of disease. *Nature Reviews Immunology*.

[29] Jang Y., Koo J. H., Kwon I. (2017). Neuroprotective effects of endurance exercise against neuroinflammation in MPTP-induced Parkinson’s disease mice. *Brain Research*.

[30] Fisher B. E., Petzinger G. M., Nixon K. (2004). Exercise-induced behavioral recovery and neuroplasticity in the 1-methyl-4-phenyl-1,2,3,6-tetrahydropyridine-lesioned mouse basal ganglia. *Journal of Neuroscience Research*.

[31] Gerecke K. M., Jiao Y., Pani A., Pagala V., Smeyne R. J. (2010). Exercise protects against MPTP-induced neurotoxicity in mice. *Brain Research*.

[32] Kintz N., Petzinger G. M., Akopian G. (2013). Exercise modifies *α*-amino-3-hydroxy-5-methyl-4-isoxazolepropionic acid receptor expression in striatopallidal neurons in the 1-methyl-4-phenyl-1,2,3,6-tetrahydropyridine-lesioned mouse. *Journal of Neuroscience Research*.

[33] Jang Y., Kwon I., Song W., Cosio-Lima L. M., Lee Y. (2018). Endurance exercise mediates neuroprotection against MPTP-mediatedParkinson’s disease via enhanced neurogenesis, antioxidant capacity, and autophagy. *Neuroscience*.

[34] Ahmad S. O., Park J. H., Stenho-Bittel L., Lau Y. S. (2009). Effects of endurance exercise on ventral tegmental area neurons in the chronic 1-methyl-4-phenyl-1,2,3,6-tetrahydropyridine and probenecid-treated mice. *Neuroscience Letters*.

[35] Paxinos G., Franklin K. (2013). *The Mouse Brain in Stereotaxic Coordinates*.

[36] Abercrombie M. (1946). Estimation of nuclear population from microtome sections. *The Anatomical Record*.

[37] Afzalpour M. E., Chadorneshin H. T., Foadoddini M., Eivari H. A. (2015). Comparing interval and continuous exercise training regimens on neurotrophic factors in rat brain. *Physiology & Behavior*.

[38] Pałasz E., Bąk A., Gąsiorowska A., Niewiadomska G. (2017). The role of trophic factors and inflammatory processes in physical activity-induced neuroprotection in Parkinson’s disease. *Postepy higieny i medycyny doswiadczalnej*.

[39] Baquet Z. C., Bickford P. C., Jones K. R. (2005). Brain-derived neurotrophic factor is required for the establishment of the proper number of dopaminergic neurons in the substantia nigra pars compacta. *Journal of Neuroscience*.

[40] Chandra G., Roy A., Rangasamy S. B., Pahan K. (2017). Induction of adaptive immunity leads to nigrostriatal disease progression in MPTP mouse model of Parkinson’s disease. *Journal of Immunology*.

[41] Schwenkgrub J., Zaremba M., Joniec-Maciejak I., Cudna A., Mirowska-Guzel D., Kurkowska-Jastrzębska I. (2017). The phosphodiesterase inhibitor, ibudilast, attenuates neuroinflammation in the MPTP model of Parkinson’s disease. *PLoS One*.

[42] Smeyne R. J., Breckenridge C. B., Beck M. (2016). Assessment of the effects of MPTP and paraquat on dopaminergic neurons and microglia in the substantia nigra pars compacta of C57BL/6 mice. *PLoS One*.

[43] Pothakos K., Kurz M. J., Lau Y.-S. (2009). Restorative effect of endurance exercise on behavioral deficits in the chronic mouse model of Parkinson’s disease with severe neurodegeneration. *BMC Neuroscience*.

[44] De Lau L. M. L., Breteler M. M. B. (2006). Epidemiology of Parkinson’s disease. *The Lancet Neurology*.

[45] al-Jarrah M., Pothakos K., Novikova L. (2007). Endurance exercise promotes cardiorespiratory rehabilitation without neurorestoration in the chronic mouse model of parkinsonism with severe neurodegeneration. *Neuroscience*.

[46] Koo J.-H., Cho J.-Y., Lee U.-B. (2017). Treadmill exercise alleviates motor deficits and improves mitochondrial import machinery in an MPTP-induced mouse model of Parkinson’s disease. *Experimental Gerontology*.

[47] O’Dell S. J., Gross N. B., Fricks A. N., Casiano B. D., Nguyen T. B., Marshall J. F. (2007). Running wheel exercise enhances recovery from nigrostriatal dopamine injury without inducing neuroprotection. *Neuroscience*.

[48] Zigmond M. J., Smeyne R. J. (2014). Exercise: is it a neuroprotective and if so, how does it work?. *Parkinsonism & Related Disorders*.

[49] Howells D. W., Porritt M. J., Wong J. Y. F. (2000). Reduced BDNF mRNA expression in the Parkinson’s disease substantia nigra. *Experimental Neurology*.

[50] Chauhan N. B., Siegel G. J., Lee J. M. (2001). Depletion of glial cell line-derived neurotrophic factor in substantia nigra neurons of Parkinson’s disease brain. *Journal of Chemical Neuroanatomy*.

[51] Cotman C. W., Berchtold N. C. (2002). Exercise: a behavioral intervention to enhance brain health and plasticity. *Trends in Neurosciences*.

[52] Hennigan A., O'Callaghan R. M., Kelly Á. M. (2007). Neurotrophins and their receptors: roles in plasticity, neurodegeneration and neuroprotection. *Biochemical Society Transactions*.

[53] Hou L., Chen W., Liu X., Qiao D., Zhou F. M. (2017). Exercise-induced neuroprotection of the nigrostriatal dopamine system in Parkinson’s disease. *Frontiers in Aging Neuroscience*.

[54] Boger H., Middaugh L., Huang P. (2006). A partial GDNF depletion leads to earlier age-related deterioration of motor function and tyrosine hydroxylase expression in the substantia nigra. *Experimental Neurology*.

[55] Gerecke K. M., Jiao Y., Pagala V., Smeyne R. J. (2012). Exercise does not protect against MPTP-induced neurotoxicity in BDNF happloinsufficent mice. *PLoS One*.

[56] Wu S. Y., Wang T. F., Yu L. (2011). Running exercise protects the substantia nigra dopaminergic neurons against inflammation-induced degeneration via the activation of BDNF signaling pathway. *Brain, Behavior, and Immunity*.

[57] Real C. C., Ferreira A. F. B., Chaves-Kirsten G. P., Torrão A. S., Pires R. S., Britto L. R. G. (2013). BDNF receptor blockade hinders the beneficial effects of exercise in a rat model of Parkinson’s disease. *Neuroscience*.

[58] Tansey M. G., Goldberg M. S. (2010). Neuroinflammation in Parkinson’s disease: its role in neuronal death and implications for therapeutic intervention. *Neurobiology of Disease*.

[59] Vivekanantham S., Shah S., Dewji R., Dewji A., Khatri C., Ologunde R. (2015). Neuroinflammation in Parkinson’s disease: role in neurodegeneration and tissue repair. *International Journal of Neuroscience*.

[60] Chen L.-W., Dong M.-H., Kuang F., Liu J.-T., Zhang J.-Q., Bai Y. (2015). Microglia and astroglia: the role of neuroinflammation in lead toxicity and neuronal injury in the brain. *Neuroimmunology and Neuroinflammation*.

[61] Neal M., Richardson J. R. (2018). Epigenetic regulation of astrocyte function in neuroinflammation and neurodegeneration. *Biochimica et Biophysica Acta - Molecular Basis of Disease*.

[62] Little A. R., O’Callaghan J. P. (2001). Astrogliosis in the adult and developing CNS: is there a role for proinflammatory cytokines?. *Neurotoxicology*.

[63] Choi S. S., Lee H. J., Lim I., Satoh J. I., Kim S. U. (2014). Human astrocytes: secretome profiles of cytokines and chemokines. *PLoS One*.

[64] Swiątkiewicz M., Zaremba M., Joniec I., Członkowski A., Kurkowska-Jastrzębska I. (2013). Potential neuroprotective effect of ibuprofen, insights from the mice model of Parkinson’s disease. *Pharmacological Reports*.

[65] Łabuzek K., Skrudlik E., Gabryel B., Okopień B. (2015). Anti-inflammatory microglial cell function in the light of the latest scientific research. *Annales Academiae Medicae Silesiensis*.

[66] McGeer P. L., McGeer E. G. (2008). Glial reactions in Parkinson’s disease. *Movement Disorders*.

[67] Saavedra A., Baltazar G., Duarte E. P. (2007). Interleukin-1*β* mediates GDNF up-regulation upon dopaminergic injury in ventral midbrain cell cultures. *Neurobiology of Disease*.

[68] Meredith G. E., Totterdell S., Potashkin J. A., Surmeier D. J. (2008). Modeling PD pathogenesis in mice: advantages of a chronic MPTP protocol. *Parkinsonism & Related Disorders*.

[69] Mitsumoto Y., Watanabe A., Mori A., Koga N. (1998). Spontaneous regeneration of nigrostriatal dopaminergic neurons in MPTP-treated C57BL/6 mice. *Biochemical and Biophysical Research Communications*.

[70] Rothblat D. S., Schroeder J. A., Schneider J. S. (2001). Tyrosine hydroxylase and dopamine transporter expression in residual dopaminergic neurons: potential contributors to spontaneous recovery from experimental parkinsonism. *Journal of Neuroscience Research*.

[71] Cohen A. D., Zigmond M. J., Smith A. D. (2011). Effects of intrastriatal GDNF on the response of dopamine neurons to 6-hydroxydopamine: time course of protection and neurorestoration. *Brain Research*.

